# Antecedents of academic actual help-seeking behaviour and support services utilisation among distance education students

**DOI:** 10.3389/fpsyg.2024.1453321

**Published:** 2024-11-18

**Authors:** Beatrice Asante Somuah, Paul Dela Ahiatrogah, Moses Segbenya, Brandford Bervell

**Affiliations:** ^1^Department of Education, College of Distance Education, University of Cape Coast, Cape Coast, Ghana; ^2^Department of Business Studies, College of Distance Education, University of Cape Coast, Cape Coast, Ghana; ^3^Department of Mathematics and Science Education, College of Distance Education, University of Cape Coast, Cape Coast, Ghana

**Keywords:** academic actual help-seeking behaviour, antecedents, support services utilisation, distance education students, help-seeking behaviour

## Abstract

**Introduction:**

The purpose of the study was to unravel the determinants that necessitate academic help-seeking intentions and promote the utilisation of support services among distance education students.

**Methods:**

This study adopted a quantitative approach based on cross-sectional survey design. The target population of the study comprised all distance education students of the College of Distance Education, University of Cape Coast. A multi-stage sampling procedure was used to select a sample of 290 respondents for the study. The main data collection instrument was a questionnaire and the data was collected from June to December, 2023. Descriptive statistics (mean and standard deviation) were used to analyse preliminary data, while the main data was analysed by structural equation modelling technique.

**Results and discussion:**

The study found that social support, subjective needs, and availability of help were the antecedents of actual academic help-seeking behaviour among distance learners. Depressive needs were not considered as an antecedent of help-seeking behaviour. Thus, social support, subjective needs, and availability of help were significantly related to actual help-seeking behaviour among distance learners. The study further found that actual help-seeking behaviour among distance learners was also significantly related to the actual use of support services available to these learners. Finally, actual help-seeking behaviour among distance learners significantly mediated the relationship between social support, subjective needs, availability of help, and actual use of support services available to distance students. It was therefore recommended that authorities of distance education should make support services available to distance learners online and in-person to be able to meet the needs of different categories of students as well as the provisions of platforms for building social support systems among distance learners.

## Introduction

Research indicates that the success of distance education students depends on three factors: support services, logistics/administration, and learning/course materials development (Thomas and Tagler, 2019; Daudi et al., 2023). However, in addition to these elements, Bağrıacık Yılmaz and Karataş (2022) also noted students’ desire to seek help is a core element in the successful management of distance education programmes. The help-seeking behaviour is very critical in distance education due to the physical and social isolations that usually exist between the students and their institutions. Thus, academic help-seeking behaviour as a support service is also a self-regulated learning strategy in which the learner determines when help is needed and how to receive that help (Koc, 2016). In support of this assertion, Wirtz et al. (2018) stressed that when students learn and internalise knowledge, they often seek assistance from sources external to themselves. Thus, academic help-seeking is fundamental to the learning experience provided in distance education toward improving students’ learning and managing their expectations (Wirtz et al., 2018; Qayyum, 2018). It is important to note that having the feeling to seek help and actually utilising available help opportunities are not synonymous (Martin-Arbos et al., 2021; Ogan et al., 2015; Fong et al., 2023; Nagai, 2021). This assertion implies that actual help-seeking behaviour is precipitated by certain factors such as subjective needs, depressive symptoms, social support, and availability of help which would lead to the actual use of support services (Nagai, 2021; Koc, 2016; Ma et al., 2021; Wang et al., 2020). Symptoms of depression have also been found to be a significant antecedent of help-seeking intention among distance students. For instance, studies by Henze et al. (2019) and Zhu et al. (2020) found that depression needs negatively predicted help-seeking intention among distance students. Research further suggests that social support can positively influence students’ help-seeking behaviours, and help overcome barriers to seeking help, such as stigma and fear of failure (Zhu et al., 2020; Kuo and Rojewski, 2019). Finally, students’ behaviour toward seeking help is influenced by the availability of help provided by the institutions. Most students who are aware of the availability of help in their institutions are more likely to seek help when faced with academic and emotional challenges (Martín-Rodríguez et al., 2020). These phenomena warrant research into the nuances existing among the determinants of actual help-seeking behaviour and actual use of support services among distance students as disclosed by a systematic review (Daudi et al., 2023; Barker, 2007; Clough et al., 2020; Huang et al., 2021).

The lack of core support services for distance students has led to demotivation, isolation, poor performance, and attrition. Owing to these challenges, contemporary distance education institutions have implemented a number of support services for distance students (Gil-Jaurena, 2014). Nonetheless, having the feeling to seek help and actually utilising available help opportunities are not synonymous (Li et al., 2023; Gulliver et al., 2023; Duraku et al., 2023; Bryant et al., 2022; Yonemoto and Kawashima, 2023; Legros and Boyraz, 2023; Nagai, 2021). It does appear that most distance education students are not utilising the support services put in place for them and they often wander about in a helpless manner when they are confronted with situations that demand that they seek help or support (Thomas and Tagler, 2019; Daudi et al., 2023). Such behaviours are sometimes displayed through acts such as dropping out or deferring a programme without the right procedures, resuming a programme without notification, depending on peers for vital information, lack of knowledge of financial resources, low grades in some courses, and low rates in registration of courses among others (Simpson, 2016; Somuah et al., 2018; Ampofo and Somuah, 2021). The reasons for these behaviours within the context of this study remain unclear. However, Nagai (2021), Koc (2016), and Cao and Zhang (2020) reiterated that actual help-seeking behaviour is precipitated by certain factors such as subjective needs, depressive symptoms, social support and availability of help which would eventually result in the actual use of support services. To help unravel such interrelationships the following research objectives were generated to guide the conduct of the study;

1. To unravel the determinants of actual academic help-seeking behaviour among distance education students.
2. To establish the relationship between actual help-seeking behaviour and actual use of support services available to distance education students.
3. To determine how actual help-seeking behaviour mediates the relationship between availability of help, support services, depressive needs, subjective needs and actual use of support services available to distance learners.

### Theoretical framework

The study was grounded on the help-seeking theory which postulates that students usually follow a series of predictable steps to seek help for their problems. According to Sorsa et al. (2021), a help-seeking theory is a concept that explores and understands the decision-making process of help-seeking among individuals. It is a complex process characterised by problem-focused, intentional action and interpersonal interaction (Sorsa et al., 2021). The theory emphasises that help-seeking behaviour is a complex decision-making process instigated by varied problems that challenge one’s personal abilities and this makes help-seeking behaviour a multidimensional and non-linear process depending on the nature of the situation (Cornally and McCarthy, 2011; Sorsa et al., 2021). As postulated by Barker (2007), one’s ability to seek help is determined by several reasons such as individual factors; exogenous factors which include the variety of social supports available; and programme efforts and policy initiatives provided by institutions to promote help-seeking. In furtherance of the discussion of the help-seeking theory, Heerde and Hemphill (2018) commented that other existing models of help-seeking behaviour also conform to three common stages. These include problem recognition, efforts made toward acts of seeking help, and the recognition or availability of a source of help (Heerde and Hemphill, 2018). This implies that help-seeking behaviour is usually influenced by triggers such as self-realisation, subjective needs, depressive symptoms, social influence, and competing priorities among others (Heerde and Hemphill, 2018). In support, Rickwood (1995) contended that help-seeking theories can be considered as ways of building knowledge for seeking assistance, obtaining information, and gaining emotional support through adaptive and problem-focused methods. Accordingly, Hirt et al. (2020) also recognised help-seeking behaviour as one of the important self-regulated behaviours for distance education students. However, help-seeking does not occur in a vacuum, but in a complex psychosocial process in which learners must be willing to engage (Karabenick and Gonida, 2018). This theory forms the basis for the current study in investigating the complex help-seeking process based on the antecedents of actual help-seeking behaviours that encompass the overall access and utilisation of support services in distance education. The researchers perceived the process as a continuum that warranted a careful study to inform policy and practice in distance education delivery.

#### Subjective need and actual help-seeking behaviour

Experiencing tertiary education may be stressful as the learning system is entirely different from secondary schools (Daudi et al., 2023; Njoka, 2014). In addition, the transition to a more independent life, and the new learning environment as espoused in tertiary educational institutions may expose individual students to stressful conditions that may require them to make some adjustments in their physical, mental, and emotional dispositions (Bizuneh, 2021). These experiences may lead to individual students developing some subjective needs that require that they seek help. In support of this, Bok (2020) and Cao and Zhang (2020) further noted that when students become aware of their needs and purpose in life they are able to seek the needed help so as to reduce boredom and increase success in all endeavours. In a related study, Sobotka and Raman (2020) noted that there should be a broad and diversifying nature of help-seeking services in higher education due to different cultural backgrounds and personalising of the needs of students. The researchers found a significant relationship between communication apprehension and self-stigma of academic help-seeking. A similar study conducted among Chinese students by Mak and Davis (2014) reported that subjective norms had a positive and significant effect on the help-seeking behaviour of the participants. The implication is that there is a need for institutions of higher learning to create multiple help-seeking services and opportunities that would encourage distance education students in particular to open up on their subjective needs that relate to academic issues. Subjective needs of a distance learner in this study will consider support systems such as the desire for emotional and academic support, including access to instructors, peers, and mentoring. It will also include flexibility such as the need for a flexible schedule that accommodates personal and professional commitments. The subjective needs will also consider access to resources among distance learners such as the need for easy access to learning materials, technology, and online resources. In support of the foregoing views, the current study hypothesised that;

*H1*: There is a statistically significant positive relationship between subjective needs and actual help-seeking behaviour of distance education students.

#### Depressive symptoms and actual help-seeking behaviour

There are generally a variety of stressful events that students face in their personal and academic lives and depending on the intensity of such situations, such students may develop some depression symptoms (Lulu et al., 2022). Some distance education students are also likely to experience depression symptoms due to isolation and lack of social connections. These symptoms could lead to withdrawal and other related anti-social behaviours (Liu et al., 2022). Such instances further create the need for students to develop help-seeking behaviours to enable them to overcome and deal with depression symptoms and their associated defects. A study by Mirhosseini et al. (2022) reported the prevalence of depressive symptoms among students when they experienced critical conditions such as a pandemic. In a related study, Wagner et al. (2022) also found that 10% of the participants developed depressive symptoms as a result of their inability to meet the needed academic requirements for progression. Daly (2022) further asserted in dealing with depressive symptoms, issues of gender should not be overlooked. This is because some studies have revealed female participants reported a higher risk of depression as compared to their male counterparts (Daly, 2022; Barra, 2019). To test for the existence of the relationship between these variables, the current study hypothesised that;

*H2*: There is a statistically significant positive relationship between depressive symptoms and actual help-seeking behaviour of distance education students.

#### Social support and actual help-seeking behaviour

In any academic environment, there occur situations when students may find it difficult to cope with some socio-educational situations and academic challenges because they go beyond their competencies, capabilities, aptitudes and perceptions (Dueñas et al., 2021) In such instances, students are likely to develop the behaviours to seek help. In furtherance of this assertion, Schlusche et al. (2021) contended that while adapting to an academic environment, students also face cognitive and social challenges and their peers sometimes act as social support to help such students develop the needed behaviours to seek help (Li et al., 2023; Schlusche et al., 2021). Thus, through such behaviours, students are able to obtain helpful guidance, and recognition from peers as well as gain expertise in the development of relationships. In connection with this assertion, a study by Dueñas et al. (2021) found significant relationships between socio-emotional variables, namely functional social support, life satisfaction, happiness, and social connections and behaviours toward help-seeking. The authors concluded that as social support increased, the behaviours of students to seek help also developed. In another instance, Qayyum’s (2018) investigation delved into attitudinal factors that impelled students to solicit assistance from both their peers and instructors. The study revealed that respondents exhibited a preference for seeking help from their classmates as opposed to their instructors (Qayyum, 2018). It was further discovered that the disposition to work independently was a significant predictor in determining whether students sought help from their instructors outside of class. It is in line with the above discussion that the current study sought to test the understated hypothesis;

*H3*: There is a statistically significant positive relationship between social support and actual help-seeking behaviour of distance education students.

#### Availability of help and actual help-seeking behaviour

Help-seeking behaviours are conscious efforts made by students to communicate an emotional pain, a problem or a mental issue in order to obtain support, a piece of advice, or help that would result in the reduction of such distressful situations and also promote academic success (Li et al., 2023; White et al., 2018). In addition, one of the conditions that can precipitate a student’s behaviour to seek help is the knowledge or exposure to the availability of help that is suitable enough to resolve personal or academic issues. Like their counterparts in traditional university settings, distance education students are prone to various psychological conditions such as anxiety, depression, interpersonal issues, and romantic relationship difficulties as noted by Asante and Andoh-Arthur (2015) and David and Roberts (2021). However, as opined by Li et al. (2023), the majority of undergraduate students do not have good knowledge about the availability of services when faced with a new challenge. Additionally, Newman (2008) mentioned that the learning environment where students find themselves coupled with the types of resources available for help-seeking has a great influence on students’ behaviour of help-seeking. A related study Craig (2023), also found that the academic progress of students was affected in a variety of ways by the existence and quality of services at the university. A similar study by Micari and Calkins (2021) also showed that, when students have positive help-seeking behaviours due to the availability of help, they progress smoothly in their course of study. In order to confirm whether a relationship existed between the availability of help and actual help-seeking behaviour, this study hypothesised that;

*H4*: There is a statistically significant positive relationship between the availability of help and actual help-seeking behaviour of distance education students.

#### Actual help-seeking behaviour and actual use of support services

Some scholars have argued that though students may possess positive help-seeking behaviours toward the support services provided by their institutions, most students prefer to approach friends, family, or academic staff when faced with conditions that demand that they seek help (Goodwin et al., 2016; Hughes et al., 2018). These trends suggest that help-seeking behaviours are a complex phenomenon that could be affected by factors such as prior knowledge, self-regulation and academic goal orientations and influence the extent of support service usage among students (Koc, 2016; Broglia et al., 2021; Molla, 2022). In support of this argument, Onyango and Aloka (2018) and Fittrer (2016) reiterated academic help-seeking behaviour is not only elemental for success in academic performance but is an important skill for lifelong education careers among students. As noted by Molla (2022), students with strong help-seeking behaviours could be potential users of support services. It is in line with this argument that the cultivation of actual help-seeking behaviours and actual use of support services among college students cannot be undervalued Focusing on the use of support services, a study by Çebi and Demir (2020) revealed that the participants most frequently used friends as sources of information and help despite the availability of support services provided by their institution. In a related study, Haile et al. (2020) found that female respondents showed more favourable help-seeking behaviours and had positive attitudes toward the actual use of support services than their male counterparts. These findings suggested further investigations into the nuances of actual help-seeking behaviour and actual use of support among university students in distance education programmes. Therefore the current study tested the following hypotheses to establish the extent of their relationships by indicating that;

*H5*: There is a statistically significant positive relationship between actual help-seeking behaviour and actual use of support services by distance education students.

*H6*: Actual help-seeking behaviour significantly mediates the relationship between social support and the actual use of support services.

*H7*: Actual help-seeking behaviour significantly mediates the relationship between subjective need and actual use of support services.

*H8*: Actual help-seeking behaviour significantly mediates the relationship between the availability of help and the actual use of support services.

*H9*: Actual help-seeking behaviour significantly mediates the relationship between depressive symptoms and the actual use of support services.

### Conceptual model

**Figure 1:**
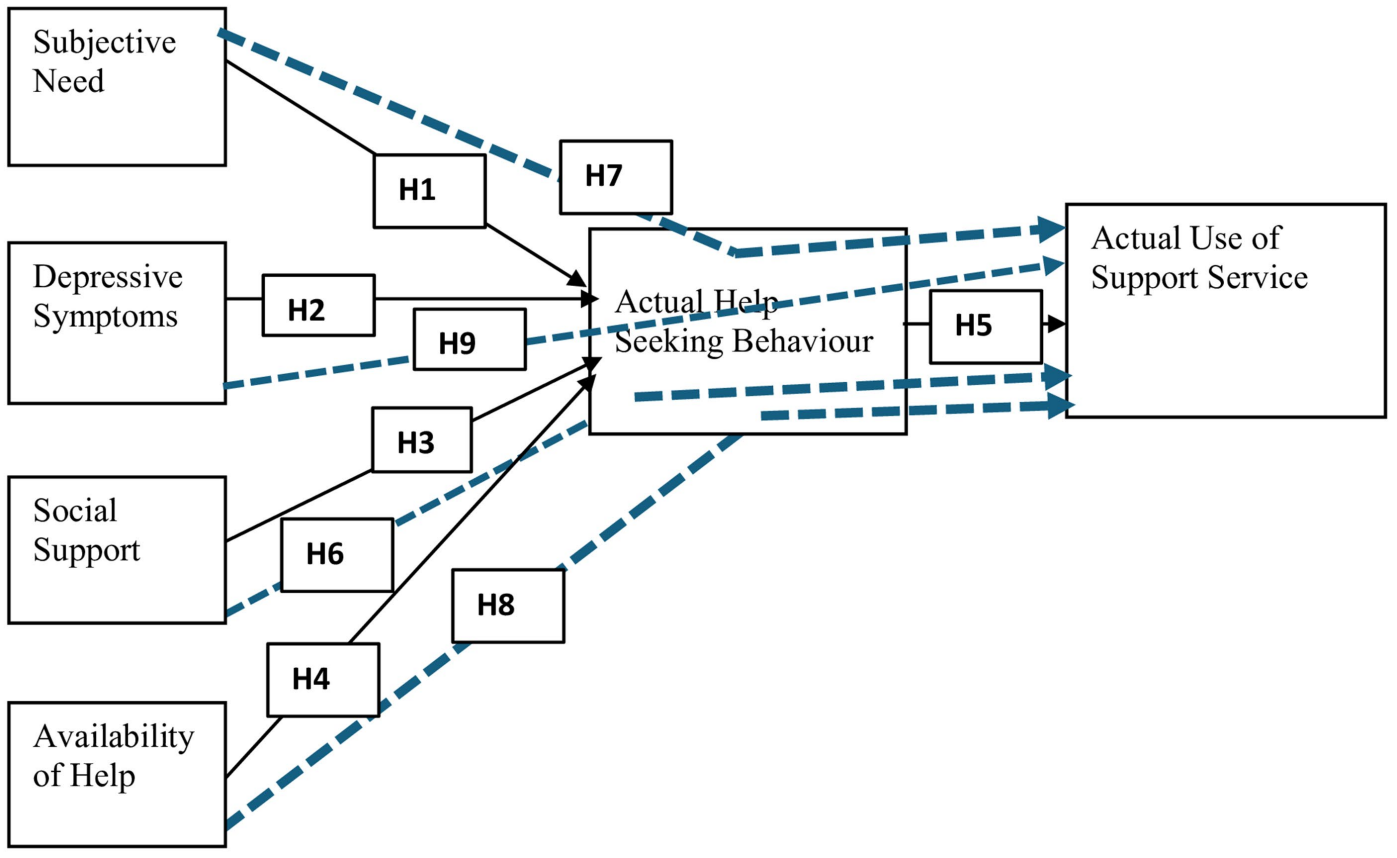
Proposed Academic Help-seeking Model in Distance Education (PAHMDE). ALT TEXT: Six circle images representing the antecedents of actual use of support services provided for distance education students. The relationship that exists between these variables is also depicted by arrows. 
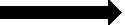
 Represent Direct relationship. 
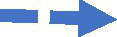
 Represent indirect relationship/mediating role. Source: Authors’ constructs based on the review of literature.

Based on the formulated hypotheses, the study proposes a conceptual model as seen in Figure 1.

## Methodology

This study was guided by a cross-sectional survey and correlational research design. A cross-sectional survey design was used for the purposes of helping the researchers to systematically collect one-time data across all the academic programmes, gender, and age of distance students in a public university in Ghana (Creswell and Creswell, 2018; Segbenya and Anokye, 2023). The correlational perspective also helped to establish a relationship between the exogenous and endogenous variables of the study. One kind of non-experimental research technique for analysing the connections between two or more variables is correlational study design. These are the main characteristics and elements of the design of the correlational study. Instead of establishing cause-and-effect links, the main goal is to identify and assess the strength and direction of relationships between variables. Out of about 21,893 distance education students across the country, 290 samples were drawn for this study. Though the study estimated 327 samples based on the suggestion (Krejcie and Morgan's, 1970; Segbenya and Minadzi, 2023; Segbenya et al., 2023) sample determination table, only 290 responses were valid to be used for the analysis at the end of the data collection. The 290 sample therefore represented an 89 percent response rate from the 327 sample estimated for this study. To qualify (inclusion criteria) to be included in this study as a respondent, one must be a distance learner with a distance education institution in a developing economy and one must have academic needs for which help-seeking becomes very relevant. Thus, a non-distance learner is excluded from this study (exclusion criteria). The study adopted simple random and stratified sampling techniques. The simple random sampling technique was used to ensure that every respondent in this study had an equal chance of being selected. The stratified sampling technique was also used to ensure that all strata such as gender, level of programmes, and location of study centre among others are considered in selecting respondents for the study. Thus, respondents for this study cut across all these strata in the population.

A questionnaire was used for the data collection across the sixteen regions of Ghana and the data was collected from June to December, 2023. Items used for measuring subjective needs were adopted from Daudi et al. (2023), and items for Depressive Symptoms were adopted from Singh et al. (2023). Also, items for Social Support and Actual Help-seeking Behaviour were adopted from Dueñas et al. (2021) and items for the Availability of help were also adopted from Li et al. (2023). Finally, items for Actual use of support services were also adopted from Molla (2022). The questionnaire was divided into two main parts. Part one was on the demographic characteristics of respondents and part two was on the objectives and hypotheses of the study. The questionnaire was also measured on a four-point Likert scale. This study avoided using the neutral as part of the scale since the researchers expected every respondent who agreed to participate in this study to make a choice of either agreeing or disagreeing with the items available. This explains why the usual five-point Likert scale was reduced to a four-point Likert scale of measurement.

The results of Cronbach’s Alpha presented in Appendix A revealed that the items referring to each variable homogeneously measure the concept under study and that the fidelity coefficient is excellent in the variable-Subjective Needs, Social Support, Actual Help-Seeking Behaviour, Availability of Help and Actual Use of Support Services, as they present values between 0.85 and 0.95.

In conducting this study, ethical considerations were made ensuring that all participants had the freedom to either participate or withdraw from the studies even if they had commenced the process. The study also ensured that participants did not suffer from any physical or psychological harm as well as ensuring confidentiality and anonymity for the responses given. The ethical clearance for this study was granted by the Institutional Review Board of the University of Cape Coast with the ID (UCCIRBEXT/2023/27) indicating that researchers were permitted to carry out this study involving human subjects. The data collected were analysed with Partial Least Square-Structural Equation Modelling. Meanwhile, the demographic characteristics of respondents were analysed with descriptive statistics such as frequencies and percentages.

### Results for hypotheses testing

This section presents results based on the hypotheses guiding the study. However, before the results are presented, the demographic characteristics of respondents to guide the reader were presented in Table 1. The key demographic characteristics were gender, age, level, and category of academic programmes among others. It is clear from Table 1 that most of the respondents were male students (61.7%), level 400 or final year students (60.0%), pursuing education programmes (78.6%); were 28–32 years (33.1%). Also, it is clear from Table 1 that the majority of the respondents were located in the Southern Zone enclave of the country.

**Table 1:** Demographic characteristics of respondents.

Demographic characteristics	No	%
Gender		
Male	179	61.7
Female	111	38.3
Total	290	100.0
Level of programmes		
Level 100	27	9.3
Level 200	22	7.6
Level 300	54	18.6
Level 400	174	60.0
Others	13	4.5
Category of programme		
Education	228	78.6
Business	50	17.2
Maths, science and ICT	12	4.1
Total	290	100.0
Age		
18–22	7	2.4
23–27	86	29.7
28–32	96	33.1
33–37	74	25.5
38–42	18	6.2
43 and above	9	3.1
Total	290	100.0
Zone/location of the respondent		
Southern Zone	240	82.8
Northern Zone	50	17.2
Total	290	100.0

Source: Field survey (2023).

**Figure 2:**
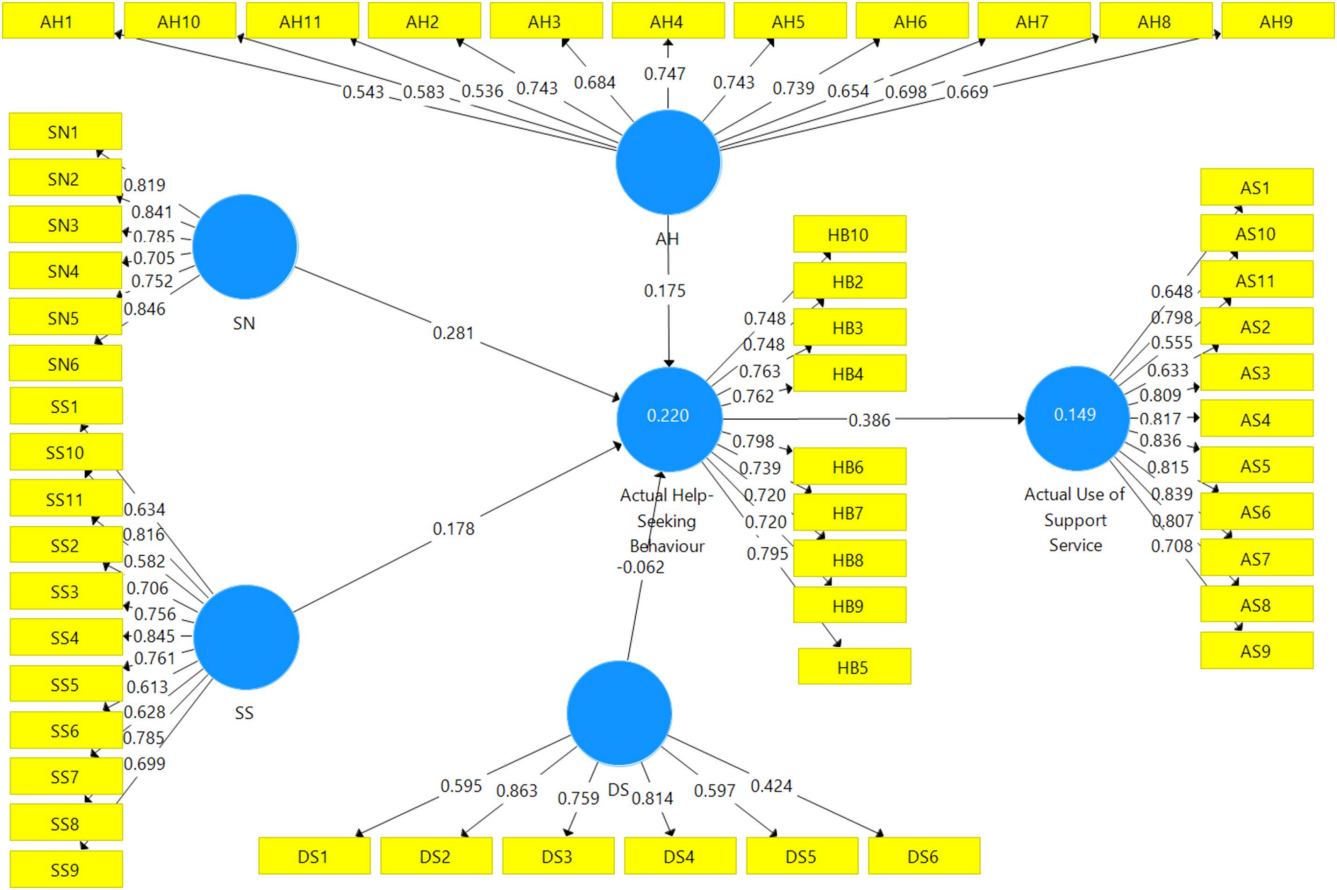
CFA algorithm. Source: Field survey (2023).

The results for the main hypotheses were also preceded by preliminary analysis to check the internal consistencies of the PLS-SEM model used for the study. The first preliminary analysis conducted was to check how items were loaded for each of the variables used for the study. The threshold used was an item loading of 0.70 according to Hair et al. (2017). The results presented in Figure 2 present all items measuring each of the variables of the study. It can be seen from Table 2 that some items loaded below the minimum threshold of 0.70. Thus, these items were deleted based on the suggestion of Hair et al. (2017).

**Table 2:** Construct reliability and validity.

	rho_A	Composite reliability	Average variance extracted (AVE)	Cronbach’s alpha
AH	0.800	0.865	0.616	0.792
HB	0.910	0.923	0.570	0.906
AS	0.932	0.941	0.668	0.928
DS	0.838	0.876	0.702	0.790
SN	0.906	0.910	0.629	0.883
SS	0.898	0.916	0.647	0.889

Source: Field survey (2023).

**Figure 3:**
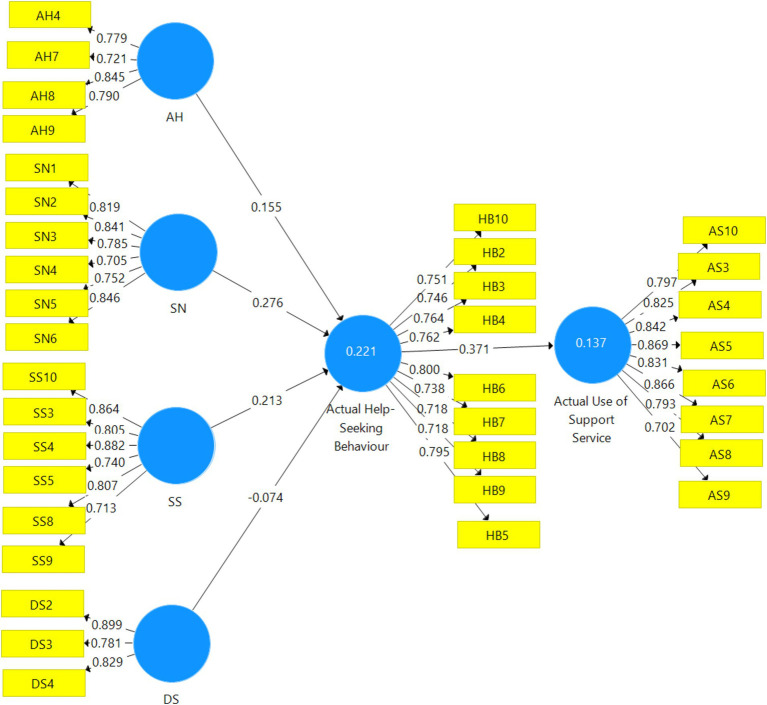
EFA of the algorithm. Source: Field survey (2023).

The revised version of Figure 2 is therefore presented in Figure 3 showing only items that loaded 0.70 and above based on the criteria used for this study. It is therefore clear that four acceptable items measured AH (availability of help), six items for SN (subjective needs) and SS (social support), and three items for DS (depressive need) variables of the study. It is also clear that nine and eight items, respectively, measured the HS (actual help-seeking behaviour) and AS (actual use of support service) variables of the study.

The next internal consistency check was done to ascertain the reliability and validity of the variables under investigation and the results are therefore presented in Table 2. The four main indices used to check the reliability and validity were rho_A, Composite Reliability, Cronbach’s Alpha and Average Variance Extracted (AVE). The minimum threshold established for the first three indices used was 0.70 based on the suggestion of Hair et al. (2017). The results presented in Table 3 revealed that rho_A recorded values between 0.800 and 0.932, and Composite Reliability also recorded values between 0.865 and 0.941. The Cronbach’s Alpha also recorded values ranging from 0.790 to 0.906. These values recorded for the first three indices were all above the recommended minimum thresholds suggesting that the variables met the reliability and validity criteria under the first three indices. The last indicator which was the Average Variance Extracted (AVE) was also based on the minimum threshold of 0.50 recommended by Segbenya et al. (2022). The AVE therefore recorded values ranging from 0.570 to 0.702 suggesting that all the variables also attained the reliability and validity requirement. Thus, the PLS-SEM model used for this study attained the reliability and validity required and could be used for further analysis.

**Table 3:** Heterotrait-monotrait ratio and collinearity (HTMT).

	AH	HB	AS	DS	SN	SS
AH	0					
HB	0.312	0				
AS	0.433	0.389	0			
DS	0.204	0.199	0.139	0		
SN	0.221	0.385	0.126	0.156	0	
SS	0.310	0.345	0.381	0.249	0.214	0
Inner VIF values						
AH		1.107				
HB			1.000			
AS						
DS		1.061				
SN		1.066				
SS		1.120				

Source: Field survey (2023).

### Discriminant validity

The discriminant validity of this study was checked with Heterotrait-Monotrait Ratio (HTMT) and the results were presented in Table 3. The maximum cut-off point was 0.850 suggested by Ringle et al. (2015). The results in Table 3 revealed that all values reported were below the maximum thresholds suggesting that the PLS-SEM used for this study attained the discriminant value status as such the variables of the study were distinct from each other.

The last internal consistency checked for the PLS-SEM used for the study was for the presence of multicollinearity (using the inner VIF) which influenced the path relation results yet to be ascertained. The maximum threshold of 3.0 suggested by Hair et al. (2017) was used to recommend that values above these thresholds suggest the presence of multicollinearity and could influence the yet-to-be-established results for path relations. The values reported in Table 3 were all between 1.000 and 1.107 which were far below the maximum threshold. This means that the PLS-SEM model used did not have any presence of multicollinearity.

### Results for hypothèses tested

Nine hypotheses guided the analysis in this section. The first five hypotheses were on the direct relationship between the six variables of the study. The second part of the results also focuses on how the actual help-seeking behaviour mediates the relationship between the remaining five variables of the study. The results for the hypotheses tested are presented in Table 4. The results presented in Table 4 revealed that four out of the first five direct hypotheses were accepted because they attained statistically significant status. Additionally, three out of the last four hypotheses were also accepted because they also attained a statistically significant status. Specifically, there was a significant relationship between subjective need (SN) and actual help-seeking behaviour (HB) at (*β* = 0.276, *t* = 4.005, *p* < 0.000) for the first hypothesis. Social support (SS) was also significantly related to actual help-seeking behaviour (HB) (*β* = 0.213, *t* = 3.813, *p* < 0.000) for the third hypothesis of the study. Hypotheses four and five also achieved statistically significant status because the availability of help (AH) significantly related to actual help-seeking behaviour (HB) (*β* = 0.155, *t* = 2.673, *p* < 0.008) for hypotheses four and actual help-seeking behaviour (HB) also significantly related to actual use of support services (AS) at (*β* = 0.371, *t* = 5.369, *p* < 0.000) for the fifth hypothesis. Meanwhile, hypothesis two was rejected because there was a non-significant relationship between depressive need (DN) and actual help-seeking behaviour (HB) at (*β* = 0.074, *t* = 5.361.2639, *p* = 0.207).

**Table 4:** Path coefficients.

						Confidence intervals		
	Original sample (O)	Sample mean (M)	Standard deviation (STDEV)	T statistics (|O/STDEV|)	*p* values	2.5%	97.5%	*f*^2^
1. SN - > HB	0.276	0.279	0.069	4.005	0.000	0.137	0.404	0.092
2. DS - > HB	−0.074	−0.083	0.059	1.263	0.207	−0.189	0.029	0.007
3. SS - > HB	0.213	0.215	0.056	3.813	0.000	0.110	0.319	0.052
4. AH - > HB	0.155	0.165	0.058	2.673	0.008	0.051	0.274	0.028
5. HB - > AS	0.371	0.376	0.069	5.369	0.000	0.227	0.498	0.159
Specific indirect effects								
6. SS - > HB - > AS	0.079	0.082	0.027	2.873	0.004	0.034	0.141	
7. SN - > HB - > AS	0.102	0.104	0.029	3.508	0.000	0.052	0.162	
8. AH - > HB - > AS	0.057	0.062	0.026	2.254	0.025	0.019	0.115	
9. DS - > HB - > AS	−0.027	−0.031	0.023	1.209	0.227	−0.075	0.011	
	R square					R square adjusted		
HB	0.221					0.210		
AS	0.137					0.134		

In terms of the indirect effect, the results in Table 4 revealed that actual help-seeking behaviour (HB) significantly mediated the relationship between social support (SS) and actual use of support services (AS) at (*β* = 0.079, *t* = 2.873, *p* < 0.004) for hypothesis six. The seventh hypothesis was also accepted because actual help-seeking behaviour (HB) significantly mediated the relationship between subjective need (SN) and actual use of support services (AS) at (*β* = 0.102, *t* = 3.508, *p* < 0.000). The last significant hypothesis was hypothesis eight which was also accepted because actual help-seeking behaviour (HB) significantly mediated the relationship between the availability of help (AH) and actual use of support services (AS) at (*β* = 0.057, *t* = 2.254, *p* < 0.025). Meanwhile, the last hypothesis of the study (Hypothesis nine) was rejected because actual help-seeking behaviour (HB) non-significantly mediated the relationship between depressive needs (DS) and actual use of support services (AS) at (*β* = 0.027, *t* = 1.209, *p* < 0.227).

Apart from the individual contribution of the six variables of the study presented with the beta values, it is also important to note that the overall contribution in explaining the variance in the actual help-seeking behaviour (HB) was 22 percent represented by the R-square and supported with the adjusted R-square. The overall contribution of the model in explaining the variance in the actual use of support services (AS) was approximately 14 percent.

**Figure 4:**
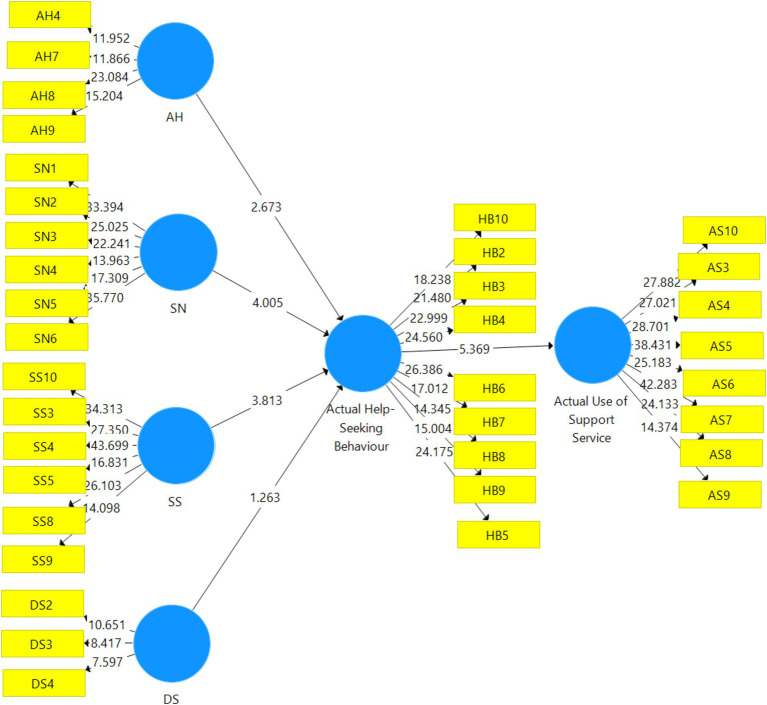
Bootstrapping results for the study.

The significant and non-significant results on the relationship between the variables of the study are further supported by the pictorial view of the interconnectedness between the variables in Figure 4. Thus, the results in Figure 4 are a confirmation that there were significant and non-significant relationships between the variables of the study.

## Discussion of findings

The findings for the first hypothesis of the study that subjective needs significantly related to actual help-seeking behaviour meant that any percentage increase in subjective needs of distance learners would result in the same percentage increase in actual help-seeking behaviour. The results meant that distance learners’ needs can best be addressed if they develop the behaviour of seeking the appropriate help from the right sources or units in the university. This further meant that it was not enough to have a need as distance learners but the right attitude and behaviour on the part of the learner are also required for such challenges to be addressed. Thus, the assertion by Heerde and Hemphill (2018) is based on the help-seeking theory that one’s ability to seek help is determined by several reasons such as individual factors and exogenous factors is upheld by the outcome of this study. The findings of this study further confirm the findings of Bok (2020) and Sobotka and Raman (2020) who reiterated that that the right behaviour is required by learners to address their subjective needs as help-seeking behaviours are also self-regulated skills to be developed by distance learners.

The findings for hypothesis two that depressive needs had a negative and non-significant relationship with actual help-seeking behaviour meant that distance learners’ subjective needs could gravitate to a higher level which may be characterised by mood disorders, feelings of sadness, loss, or anger that might interfere with their learning activities. Therefore any percentage increase in depressive needs of these learners would not result in the same percentage increase in actual help-seeking behaviour among distance learners. This could mean that distance learners could refrain from seeking help if they realise that their challenges have persisted for a long and have aggravated without any means of resolving them. Thus, students might give up on prolonged challenges and might not seek any academic help from their institutions. Thus Heerde and Hemphill's (2018) position was based on the help-seeking theory that help-seeking was influenced by three steps such as problem recognition, efforts made toward acts of seeking help, and the recognition or availability of a source of help was supported by the findings of this study. The findings of this study supported other studies by Lulu et al. (2022), Singh et al. (2023), and Mirhosseini et al. (2022) who found that depressive needs significantly influenced actual help-seeking behaviour. The results meant that if learners with depressive needs did not seek help, then the possible effects on their academic and personal lives could be devastating.

Social support was also found to be significantly related to actual help-seeking behaviour among distance learners (hypothesis three). This meant that any percentage increase in the availability of social support would also result in the same percentage increase in actual help-seeking behaviour among distance learners. The implication is that the availability of special persons, family, and friends among others around the distance learners, would not only provide the impetus to seek solutions but also influence actual help-seeking behaviours among learners as stipulated in the findings of Li et al. (2023) and Schlusche et al. (2021). That meant that as much as the close people around the distance learner were very important in providing social support in times of need, these stakeholders could also influence the behaviour of distance learners to seek for better solution from the right sources if they were not able to provide those solutions themselves. Thus, the reliability of the social support system around the distance learner can lead to help-seeking behaviour among distance learners as confirmed by Qayyum (2018). The conclusion of the help-seeking theory that there were exogenous factors that influenced help-seeking behaviour is therefore confirmed by the findings of this study (Baker, 2007).

The findings for hypothesis four that the availability of help significantly relates to actual help-seeking behaviour meant that the knowledge of the availability of help for distance learners was required to stimulate help-seeking behaviour among distance learners. The results also suggested that a lack of knowledge of the availability of help to the academic needs of distance learners would curtail their help-seeking behaviour. Thus, the need for academic institutions to make known the type or nature of support services available to distance learners and how to access such support services during orientation programmes could be very helpful in this regard. The results gave credence to the importance of the third stage of the help-seeking behaviour theory which was indicated as the recognition or availability of a source of help (Heerde and Hemphill, 2018). These findings further support the findings of Micari and Calkins (2021) and Daudi et al. (2023) that actual help-seeking behaviour was influenced by the availability of help for learners. Thus authorities of distance education institutions seeking to influence help-seeking behaviours among distance learners would therefore need to make help available and also ensure that distance learners were very much aware of the existence of such help for easy accessibility.

The findings for hypothesis five suggested that any percentage increase in actual help-seeking behaviour would lead to the same percentage increase in the actual use of support service as actual help-seeking behaviour was significantly related to the actual use of support service among distance learners. The results meant that until distance learners develop the culture and behaviour of seeking help it would be very difficult for such distance learners to really use the existing support services provided by their institutions. This implied that it was not enough to just institute support services for distance learners but also institutions must take an interest in putting in structures that would influence the help-seeking behaviour among learners to enable them to really use the support services available. The second stage of the help-seeking behaviour according to the help-seeking theory is therefore argued here to be the foundation for the attainment of the third stage of actually using the support services available to distance learners (Baker, 2007). The findings of the study are in agreement with the earlier findings of Koc (2016), Cao and Zhang (2020), Broglia et al. (2021), and Molla (2022) who found that actual help-seeking behaviours significantly influenced the actual use of support services available to learners.

Apart from the direct relationship established above, it was also important to take note of the indirect relationship between the variables of the study. That is, the mediating role and power that actual help-seeking behaviour had with the relationship established so far among the variables of the study. The results from hypotheses six, seven, and eight established that actual help-seeking behaviour significantly mediated the relationship between social support, subjective needs, availability of help, and actual use of support services provided by distance education institutions. The results meant that social support, subjective needs, availability of help and actual use of support shared their potency with actual help-seeking behaviours. Therefore, apart from the direct relationship established earlier, these later hypotheses further confirmed that actual help-seeking behaviours were a panacea for the actual use of support services available for distance learners. That is, it was good to provide support services to help address the subjective needs of distance learners, however, the use of these support services would be contingent upon inculcating in these students actual help-seeking behaviours. The results further meant that it was not enough for social support to be available for the distance learner, however, until a distance learner cultivated the habit of seeking help from the right sources depending on their challenges, they would not use the support services available to them. The findings of this study in relation to these three hypotheses further agreed with the works of Fittrer (2016), Onyango and Aloka (2018), Haile et al. (2020), and Li et al. (2023) that help-seeking behaviours significantly mediated the relationship between social support, subjective needs, availability of help and actual use of support among students.

The results of the last hypothesis of the study found that actual help-seeking behaviour had a non-significant mediated relationship between depressive needs and actual use of support services among distance learners in further support of hypothesis two. This meant that depressive needs did not relate to actual help-seeking behaviour and the relationship between depressive needs and actual use of support services was also not mediated by actual help-seeking behaviour. This implied that depressive needs among distance learners assumed different dimensions as compared to their subjective needs. Thus, though subjective needs had a significant relationship with actual help-seeking behaviour, the same cannot be said about depressive needs and actual help-seeking behaviour. In a similar vein, though actual help-seeking behaviour significantly mediated the relationship between subjective needs and the actual use of support services, the same cannot be said between depressive needs and the actual use of support services provided by distance education institutions.

### Theoretical and practical implications

The findings of this study have practical and theoretical implications. The first practical implication of the findings of this study was that distance learners have needs. If such needs are not well addressed, they could degenerate into depressive symptoms or other health-related challenges that were beyond the ability of the educational institutions to address them. Educational institutions, therefore, need to find a way or put in place mechanisms that address the subjective needs of distance learners. The second practical implication of the findings of this study was that a support system in terms of friends and family members among others around the distance learner was very important to either help resolve some challenges or direct the distance learner to the right source of resolution. Therefore, distance education institutions need to ensure opportunities exist for these distance learners to make friends on the programme through collaborative learning or group activities. The third practical implication of the results of this study was that every help or support service available to the distance learner could only be appropriated if the learner develops the behaviour of seeking such help. The focus of the distance education institutions seeking to support their students on how to form such behaviour of seeking the right help at the right time from the right sources. Thus, orientation and explanation for specific support services and where they can be found should be enhanced by institutions. It is important to note that the intention to seek help is not just enough to actually use the support services but rather the need for institutions to focus their attention on how to influence the behaviour of distance learners for help-seeking was the most important contribution of this study to knowledge.

The theoretical implication of this study was that emphasis had been placed on the institutions on the need to put up structures that create a conduit for the development of self-regulated behaviours or strategies among learners as a preamble to nurturing actual help-seeking behaviours toward actual use of support services. The outcome of this study further supported the help-seeking theory that distance learners also follow a series of predictable steps to seek help for their academic problems. Thus the help-seeking theory has been found to be relevant in the distance education landscape. The contribution of this study to the help-seeking theory, however, was that help-seeking intention alone was not adequate to influence policy on help-seeking among distance learners. The outcome of this study emphasised that there was a need to pay attention to the influence of the help-seeking behaviour of distance learners to achieve actual usage of support services available to learners.

## Conclusion and recommendations

This study sought to examine the antecedents of actual academic help-seeking behaviour and support services utilization among distance education students. It can be concluded that social support, subjective needs, and availability of help were the antecedents of actual academic help-seeking behaviour among distance learners. Depressive symptoms were not considered as an antecedent of help-seeking behaviour. Thus, social support, subjective needs, and availability of help were significantly related to actual help-seeking behaviour among distance learners. It could also be concluded that actual help-seeking behaviour among distance learners was also significantly related to the actual use of support services available to these learners. Finally, it could be concluded that actual help-seeking behaviour among distance learners significantly mediated the relationship between social support, subjective needs, availability of help, and actual use of support services available to distance students.

Based on the above conclusions, it was therefore recommended that authorities of distance education should make support services available to distance learners. These support systems could be both online and in-person to be able to meet the needs of different categories of students. It was also recommended that distance learners should be provided with platforms or mechanisms for building social support systems. This can be done by ensuring that authorities design their academic programmes in a way that encourages collaborative learning and group activities. Institutions should therefore ensure that collaborative learning and group activities are part of students’ assessment for students. It was also recommended that distance education authorities should embark on aggressive orientation among their students on the exact type of support services available to them and where and when can such services be accessed. It would be very important to have support services that are available all day around the week and the month (24/7) so that distance learners can benefit from them at their convenience.

### Suggestion for further studies

This study assumed that academic help-seeking behaviour among distance learners was a panacea for accessing support services available to these students and that having the intention to use these support services as found by earlier studies was not enough in a developing economy context. Further studies could be carried out to compare the findings of this study to what pertained to developed economies. Further studies could also consider examining other antecedents of actual help-seeking behaviour of distance learners other than the availability of help, social support, and subjective needs, among others.

## Data Availability

The raw data supporting the conclusions of this article will be made available by the authors, without undue reservation.

## Appendix A

**Table:**

Variables	Cronbach’s alpha	No of items
Subjective needs	0.881	6
Depressive symptoms	0.790	6
Social support	0.905	11
Actual help-seeking behaviour	0.888	9
Availability of help	0.876	11
Actual use of support services	0.909	10
